# The Viral Immunoshadow: Early Adenovirus Strategies for Cloaking Innate Immunity with E1A, E4orf1, and Beyond

**DOI:** 10.3390/cells15090746

**Published:** 2026-04-22

**Authors:** Marco Vezzoli, Giorgio Dieci, Roberto Ferrari

**Affiliations:** 1 Molecular and Cell Biology of the Epigenome Laboratory (MCBEL), Department of Chemistry, Life Sciences and Environmental Sustainability, University of Parma, 43124 Parma, Italy; marco.vezzoli@unipr.it (M.V.); giorgio.dieci@unipr.it (G.D.); 2 Interdepartmental Centre for Innovation in Health Products Biopharmanet-TEC, University of Parma, 43124 Parma, Italy

**Keywords:** adenovirus, E1A, e1b, e3, e4orf1, e4orf3, immunomodulation, immune evasion, epigenetic reprogramming, Alu elements

## Abstract

Human adenovirus (HAdV), a double-stranded DNA virus, targets terminally differentiated cells in the upper respiratory tract. As a key platform for gene therapy vectors, elucidating HAdV’s virulence factors is vital for optimizing therapeutic applications and mitigating risks. To achieve productive replication, HAdV strategically neutralizes host immune defenses and induces S-phase pathways essential for viral propagation. This review synthesizes the latest insights into the key pathways through which HAdVs harness these early proteins to enhance virulence, skilfully evading and counteracting host defense mechanisms while propelling viral replication. As foundational platforms for gene therapy vectors (e.g., in oncology and rare disease treatments) and vaccine backbones (e.g., COVID-19 vaccines like ChAdOx1), understanding HAdV’s immunoshadowing—the multifaceted strategies used to cloak innate and adaptive immunity—is crucial for enhancing vector safety and efficacy. Recent insights unveil how early viral proteins—including E1A, E1B-55K, E4orf1, E4orf3, E4orf6, and the E3 complex—participate in these processes. This review critically synthesizes these pathways, evaluating study limitations such as reliance on immortalized cell lines that underestimate the role of these proteins in immunological competent cells, and addresses unresolved controversies, including differential immunoshadowing efficacy across HAdV species that impacts vaccine design.

## 1. Introduction

Human adenoviruses (HAdV) are non-enveloped, double-stranded DNA viruses that typically cause self-limiting respiratory, ocular, or gastrointestinal infections in immunocompetent individuals [1,2]. Beyond their role as common pathogens, HAdVs have become one of the most versatile and clinically relevant platforms in modern biomedicine. Different HAdV serotypes exhibit distinct biological properties such as receptor usage, immunogenicity and replication efficiency, making them differentially suited for therapeutic applications [3]. For instance, species C serotypes such as HAdV-C5 have been extensively engineered as gene delivery vectors because of their efficient transduction of both dividing and non-dividing cells and high-level transgene expression [4]. However, high pre-existing humoral immunity against HAdV-C5 in the human population often limits their clinical efficacy. To circumvent this obstacle, rarer human serotypes (e.g., HAdV-B35) and non-human adenoviruses (such as chimpanzee-derived ChAdOx1, now widely used in vaccine development) have been developed as alternative vector backbones with lower seroprevalence and improved safety profiles [3].

In oncology, HAdV serotypes have been further adapted for oncolytic virotherapy. Conditionally replicative adenoviruses are designed to exploit tumour-specific defects in cell cycle control and antiviral pathways, enabling selective replication in and lysis of cancer cells while sparing normal tissue [5]. HAdV-C5-based oncolytic vectors have shown promising results in clinical trials for a variety of solid tumours, often in combination with immune checkpoint inhibitors to enhance anti-tumour immunity [5,6]. More recently, serotype-switched or chimeric vectors (e.g., incorporating fibre proteins from HAdV-B or -D serotypes) have been engineered to improve tumour targeting, reduce hepatic sequestration, and overcome pre-existing immunity, thereby broadening the therapeutic window in oncology [2,7].

These clinical successes underscore the remarkable versatility of HAdV as a therapeutic delivery vehicle. At the same time, the very properties that make HAdV such a powerful platform (efficient cell entry, robust gene expression, and the ability to evade host immunity) also represent significant challenges. Uncontrolled immune activation can lead to inflammatory toxicities, while pre-existing or rapidly developing anti-vector immunity can limit vector efficacy and durability. Therefore, a detailed mechanistic understanding of how HAdV manipulates host antiviral defences is essential not only for elucidating viral pathogenesis but also for the rational design of safer, more effective next-generation vectors, oncolytic agents, and vaccines.

To achieve productive infection, HAdV, like many other viruses, extensively remodels host cell signalling pathways and interferes with epigenetic regulators [8]. Representative examples of such viral strategies employed by diverse viruses are summarised in Table 1. Human adenoviruses are particularly sophisticated in this regard. Through the coordinated expression of their early proteins, most notably E1A, E1B, selected E4 open reading frames and the E3 immunomodulatory proteins, HAdV profoundly reprograms the host epigenome, suppresses innate immune sensing, inhibits apoptosis, and dampens adaptive immune recognition. These mechanisms, collectively referred to as “immunoshadowing”, enable the virus to create a favourable intracellular environment for replication while limiting host antiviral responses. This review focuses on the molecular strategies by which these early proteins orchestrate immunoshadowing, with particular emphasis on their interplay with epigenetic regulators and key innate and adaptive immune pathways.

## 2. The Early E1A Protein: The Architect of Genome-Wide Reprogramming

**Figure 1:**
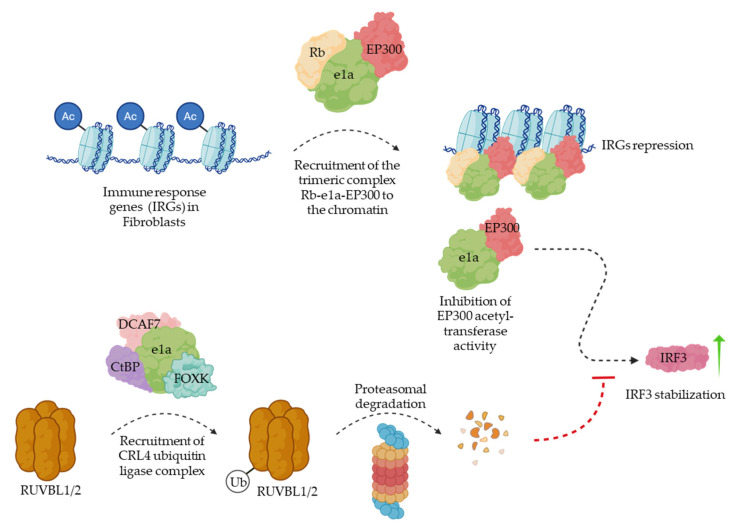
Small E1A immunoshadowing in human host cells. In the upper part, trimeric complex Rb-E1A-EP300 is shown triggering epigenetic repression of immune response gene (IRG) transcription in human fibroblasts. In the bottom part, E1A is shown to block IRF3 stabilization that would otherwise occur upon EP300 inhibition, promoting the proteasomal degradation of RUVBL1 and RUVBL2.

Small E1A acts as a molecular hub, interacting with numerous host proteins. Its N-terminal region binds the histone acetyltransferases EP300/CBP [16], the tumour suppressor RB [16], and the chromatin remodeler EP400 [16], whereas the C-terminal region interacts with FOXK [17], DCAF7 [18] and CtBP [17]. These interactions collectively suppress host antiviral responses and promote viral replication. The well-characterized interactions with EP300 and RB are responsible for extensive reorganization of the host epigenome. Binding of E1A to EP300 induces global hypoacetylation of H3K27 and H3K18 in lung fibroblasts [8,19,20,21], which in turn triggers a significant reduction in expression of genes involved in cellular immune response, IFN activation, and antigen presentation [20] (Figure 1, upper part).

Additionally, E1A sequesters RB and, together with EP300, forms a trimeric complex that is recruited to gene bodies of antiviral response genes, including those involved in TRAIL, IL-3, IL-5, and IFN-γ signalling [20] (Figure 1, upper part). Recruitment of RB-like protein p130 by this complex promotes chromatin condensation and transcriptional repression [20]. Interestingly, the EP300–E1A–RB complex enhances transcription of CXCL1, CXCL2, CXCL3, and IL-8 [20]. Although these chemokines are pro-inflammatory, they have been reported to stimulate expression of viral receptors, thereby promoting infection of neighbouring cells [22].

In addition to its N-terminal region, the C-terminal domain of E1A also engages in interactions that counteract host antiviral responses. The formation of the FOXK–DCAF7–CtBP-E1A complex recruits DCAF10, a subunit of the CUL4-RING E3 ubiquitin ligase, leading to assembly of the full CRL4 complex. The E1A–DCAF10–CRL4 complex ubiquitinates the AAA+ ATPases RUVBL1 and RUVBL2, promoting their proteasomal degradation [23] (Figure 1, lower part). Loss of RUVBL1/2 prevents stabilization of IRF3 following EP300 inhibition, thereby suppressing activation of interferon-stimulated genes [23] (Figure 1).

Beyond the repression of the cellular antiviral response, E1A interactions with RB and EP300 drive S-phase entry and host cell dedifferentiation. In dividing cells, RB–E2F disruption normally depends on cyclin–CDK-mediated RB phosphorylation in response to extracellular mitogens [24], but adenoviral E1A subverts this mechanism by acting as a mitogen and physically sequestering RB from E2F transcription factors, disrupting the RB–E2F complex and therefore inducing cell cycle progression and viral gene transcription [25]. The E1A-induced hypoacetylation of H3K27 is also associated with repression of YAP/TAZ-bound enhancers, leading to inhibition of the Hippo pathway and promoting dedifferentiation [26].

**Figure 2:**
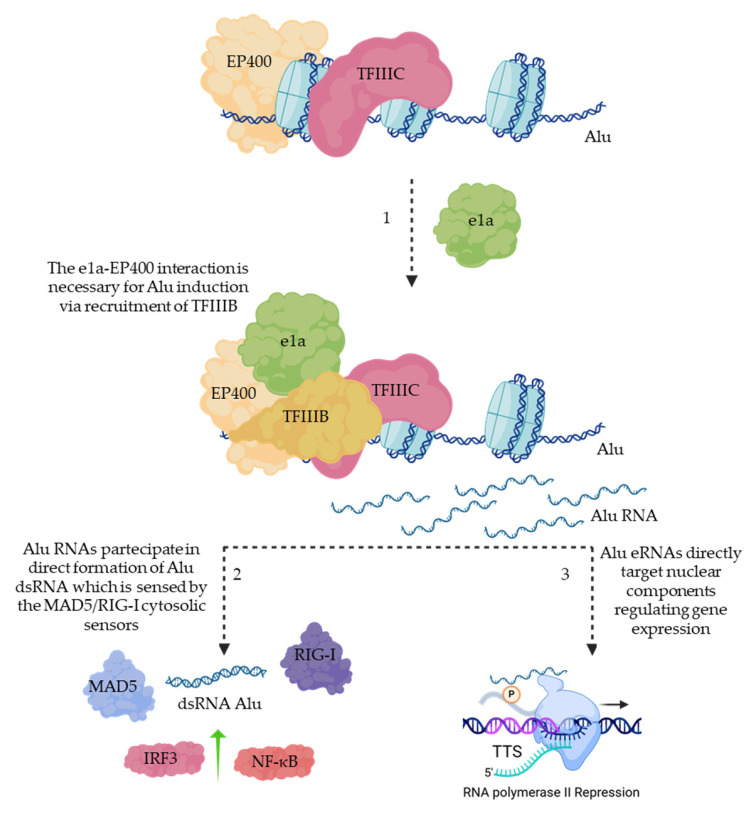
Small E1A-EP400 interaction induces transcription of Alu retrotransposons. (1) E1A is recruited on the Alu loci through the interaction with EP400, promoting the recruitment of the whole transcriptional machinery, inducing Alu transcription. Alu RNA can (2) form a dsRNA detected by the host MDA5-RIG-I, inducing an immune response, and/or (3) directly interact with the RNA polymerase II to repress transcription.

Similarly, E1A can sequester EP400 from E2F, activating E2F-dependent transcription. The E1A–p400 complex inhibits ubiquitination of MYC [27] and is recruited to MYC target gene promoters, enhancing transcription [28]. Our recent work shows that E1A localizes to genomic loci with YAP/TAZ enhancer features, bound by enhancer-associated transcription factors including YAP, TEAD4, and E1A interactors EP300, RB, and EP400 [29] (Figure 2).

Through EP400, E1A recruits TFIIIB, promoting RNA polymerase III–dependent transcription of Alu elements [29]. It is still unclear whether this Alu induction represents a proviral mechanism or a host antiviral response and further investigation will be required. However, induction of Alu transcription could lead to dsRNA activation of cytoplasmic sensors such as the MDA5 and RIG-I pathways [30,31] (Figure 2). Moreover, Alu RNAs have been shown to directly associate with RNA polymerase II, triggering its inhibition [32,33] (Figure 2).

E1A proteins from different adenovirus subgroups exhibit distinct binding preferences toward host transcriptional regulators. For example, Pelka et al. showed that E1A proteins from HAdV-3, -5, -9 and -40 interact with the pCAF/GCN5 acetyltransferase, a component of SAGA-like complexes, whereas those from HAdV-4 and -12 do not [34]. These differences suggest subgroup-specific variation in the ability to exploit chromatin-modifying machinery, which may influence downstream transcriptional reprogramming and antiviral gene expression. Separate studies focusing on the two major alternatively spliced isoforms of E1A, designated 12S and 13S, have revealed that the larger 13S isoform plays the dominant role in activating the TEAD/YAP transcriptional pathway. By stimulating TEAD/YAP activity, the 13S protein isoform promotes cell cycle progression into S phase while simultaneously inhibiting the pro-inflammatory transcription factor NF-κB in adenovirus-infected epithelial cells [26,35]. This dual action helps the virus both drive host cell DNA synthesis (which is needed for its own replication) and dampen immediate innate immune signalling. In contrast, experiments performed in physiologically relevant in vivo infection models (in this review, the term “in vivo” refers specifically to experiments performed in whole-organism models, such as murine pneumonia or humanized mouse models) show that E1A promotes immune shadowing by directly interfering with IFN-γ signalling in infected or E1A-expressing cells, thereby limiting antigen presentation to CD8^+^ cytotoxic T cells. Specifically, E1A binds the immunoproteasome subunit MECL1 and down-regulates IFN-γ-induced expression of the immunoproteasome components MECL1, LMP2, and LMP7 through reduced STAT1 phosphorylation [36].

The strengths of the aforementioned studies lie in their rigorous use of chromatin immunoprecipitation followed by sequencing (ChIP-seq), which has provided direct genome-wide evidence of E1A-induced hypoacetylation at specific enhancer regions. Nevertheless, a notable limitation is that most of these analyses have been conducted in epithelial cell models and have largely overlooked the behaviour of E1A in professional immune cells. Adenovirus E1A contributes to immune shadowing primarily by impairing antigen processing and presentation to CD8^+^ cytotoxic T cells rather than by suppressing early innate cytokine responses in alveolar macrophages. In alveolar macrophages, adenovirus internalization rapidly induces pro-inflammatory cytokine secretion, including IL-6 and TNF-α, which help initiate innate immune activation and neutrophil recruitment [37,38]. E1A’s established immunoshadowing activity occurs through direct interference with IFN-γ signalling by binding the immunoproteasome subunit MECL-1 and attenuates STAT1 phosphorylation, thereby down-regulating IFN-γ-induced expression of the immunoproteasome components MECL1, LMP2, and LMP7. This reduces MHC class I antigen presentation and limits recognition by CD8^+^ T cells, restraining adaptive effector functions without requiring complete p53 ablation [39,40]. Looking forward, a productive avenue for future research would be the generation of isoform-specific E1A knockout viruses (or conditional mutants) that allow precise dissection of how individual E1A splice variants influence cytokine networks in more sophisticated humanized mouse models. Such experiments should help to resolve ongoing debates about whether E1A’s immune evasion functions are more critical during acute lytic infections or during the establishment and maintenance of chronic/persistent adenovirus infections.

## 3. E1B-19K: Modulation of Apoptosis in the Immune Evasion of Adenoviruses

The e1b genomic region of human adenovirus encodes several proteins from a single primary transcript. Two of the most important proteins, E1B-19K and E1B-55K, are produced from distinct open reading frames rather than by alternative splicing [41]. This arrangement allows the virus to regulate the expression of E1B-19K and E1B-55K independently during the infection.

**Figure 3:**
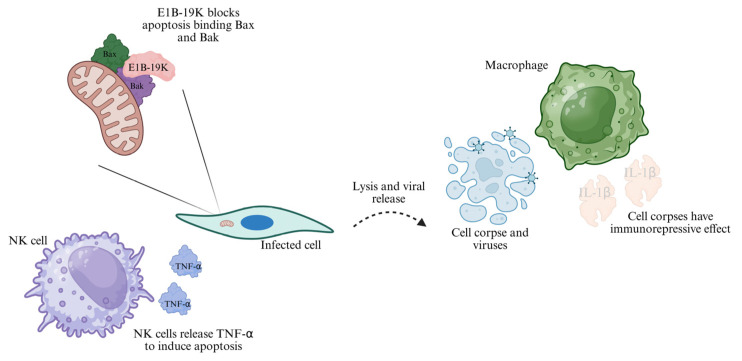
E1B-19K blocks apoptosis by binding Bax and Bak. E1B-19K binds Bax and Bak, preventing the release of cytochrome C from the mitochondria and blocking the apoptosis pathway induced by TNF-α. The cell corpses generated after the cell lysis, caused by the release of virus particles, have properties like the apoptotic corpses, causing an immune repressive effect on the macrophages.

E1B-19K is a functional homolog of the cellular anti-apoptotic protein BCL-2. It directly binds the pro-apoptotic proteins BAK and BAX, thereby preventing mitochondrial outer membrane permeabilization. This blocks the release of cytochrome c into the cytosol and subsequently inhibits caspase activation in infected cells. Such anti-apoptotic activity is essential for maintaining cell viability and sustaining viral replication even in the presence of strong innate immune signals [42,43] (Figure 3).

In immunological contexts, E1B-19K contributes to viral immunoshadowing by protecting infected cells from the TNF-α-triggered apoptosis induced by NK cells and, after the viral release, creating an immunorepressive microenvironment that mimics an apoptotic cell death, avoiding the macrophage activation and the cytokine release [43,44] (Figure 3). This protective role was clearly demonstrated by the deletion of E1B-19K, which led to markedly heightened cytokine storms (elevated IL-6 and TNF-α) and significantly reduced viral persistence in vivo [45]. However, the contribution of E1B-19K to adaptive immunity remains relatively understudied: it could indirectly support MHC class I evasion by stabilizing infected antigen-presenting cells (APCs), which in turn allows prolonged suppression of IL-12 signalling to CD4^+^ T cells determining a chronic infection [46]. Important limitations must be noted: the protein’s effects can be species-specific, because murine models do not fully recapitulate the human BCL-2–like interactions. Critically, the anti-apoptotic function of E1B-19K synergizes with proteins from the E3 region. At the same time, compensatory roles played by E4orf6 in p53 modulation illustrate functional redundancies within the viral proteome. These redundancies highlight the need for future co-deletion studies to precisely map the cytokine networks controlled by E1B-19K.

## 4. E1B-55K: Evasion by Degradation and Transcriptional Repression

**Figure 4:**
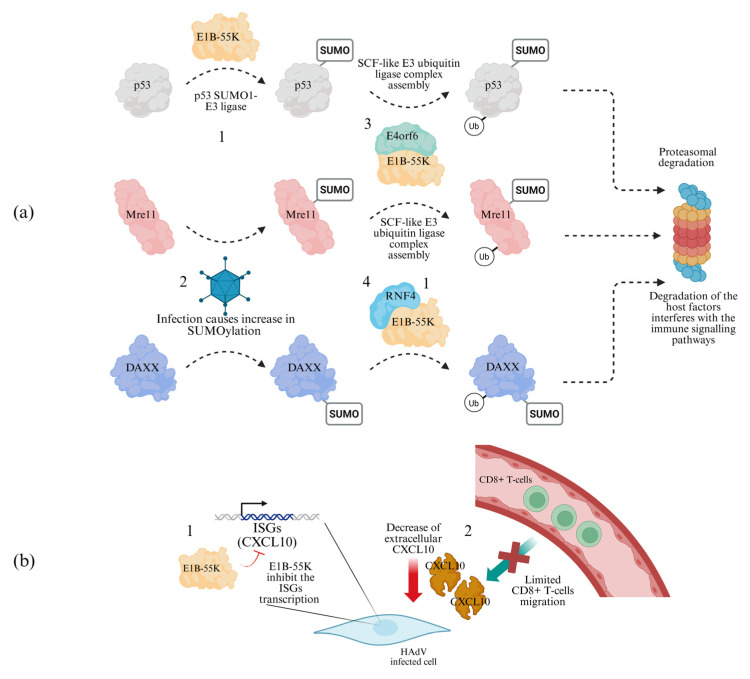
E1B-55K immunoshadowing through the degradation of antiviral host proteins. (**a**) (1, 2) Antiviral host proteins are SUMOylated in response to adenoviral infection then subsequently ubiquitinated (3) in a E1B-55K/E4orf6-dependent manner (p53, Mre11) or (4) in a E1B-55K-dependent, E4orf6-independent manner (DAXX). Ubiquitinated factors are degraded by the proteasome. (**b**) (1) E1B-55K expression prevents the transcription of the ISGs, such as CXCL10, causing its decrease in the extracellular environment. (2) The decrease in CXCL10 concentration prevents the migration of CD8^+^ T-cells to the infected tissue.

E1B-55K forms a complex with the adenoviral protein E4orf6 and host proteins Cul5, RBX1 and Elongin B/C to generate a CRL-like E3 ubiquitin ligase [47], leading to ubiquitination and degradation of p53. In addition, p53 is directly SUMOylated by E1B-55K [48] (Figure 4a). SUMO1-modified p53 exhibits reduced nuclear mobility, accumulates in promyelocytic leukaemia nuclear bodies (PML-NBs), and is subsequently exported to the cytoplasm [48].

E1B-55K SUMOylation activity extends beyond p53. Subunits of the MRN complex are SUMOylated in an E1B-55K-dependent manner and subsequently ubiquitinated by the E1B-55K/E4orf6 E3 ligase complex, leading to proteasomal degradation (Figure 4a) [49]. Other antiviral host proteins targeted through similar mechanisms include TIP60, DNA ligase IV, integrin α3, ATRX, and SPOC1 [50,51,52,53,54,55]. In addition, E1B-55K promotes ubiquitination of DAXX in an E4orf6-independent manner by recruiting the SUMO-targeted ubiquitin ligase RNF4, leading to DAXX degradation [56] (Figure 4a). E1B-55K undergoes conjugation with SUMO1 and SUMO2 at two key lysine residues: K101 (secondary site) and K104 (primary site) [57]. This SUMOylation is facilitated by the viral protein E4orf6 in cooperation with cellular E2 conjugating enzymes. The modification enhances the proteasomal targeting of DAXX and ATRX within nuclear bodies, ultimately leading to repression of IFN-β promoter activity [49]. Beyond post-translational modification, E1B-55K acts as a genome-wide transcriptional repressor of p53-associated genes by interacting with p53 at promoter regions [58]. Moreover, HAdV-C5 E1B-55K binds promoters of TEAD- and AP-1-dependent genes. Notably, E1B proteins from different adenovirus types display both shared and divergent properties. E1B-55K from the highly oncogenic HAdV-A12 shows stronger enrichment at TEAD motifs and represses TEAD-dependent transcription more efficiently than HAdV-C5 E1B-55K. Additionally, HAdV-A12 E1B-55K exhibits enrichment at CTCF binding sites, a feature not observed for HAdV-C5 E1B-55K [58]. In 2025, Dobner’s group showed that E1B-55K might modulate IFN responses in a p53-independent manner [59].These findings resolve earlier controversies regarding p53 dependency by demonstrating that E1B-55K directly silences interferon-stimulated genes (ISGs) such as IFIT1 and IFIT3. Nevertheless, important gaps remain in cytokine profiling. In vivo, E1B-55K reduces extracellular levels of the chemokine CXCL10, thereby limiting CD8^+^ T-cell migration to infected tissues [49,60,61] (Figure 4b). The strengths of these studies include robust proteomics validation of the relevant protein complexes. At the same time, there is a risk of over-interpretation when experiments rely heavily on adenovirus-transformed cell lines. Future multi-omics approaches could help clarify the role of IL-1β feedback loops in macrophages during viral dissemination. Although loss of E1B-55K SUMOylation does not impair interactions with transcription factors genome-wide, it significantly alters E1B-55K chromatin binding profiles [62].

## 5. E3 Proteins: Key Players in Adaptive Immune Evasion

The E3 region is transcribed early after infection and encodes a suite of immunomodulatory proteins.

**Figure 5:**
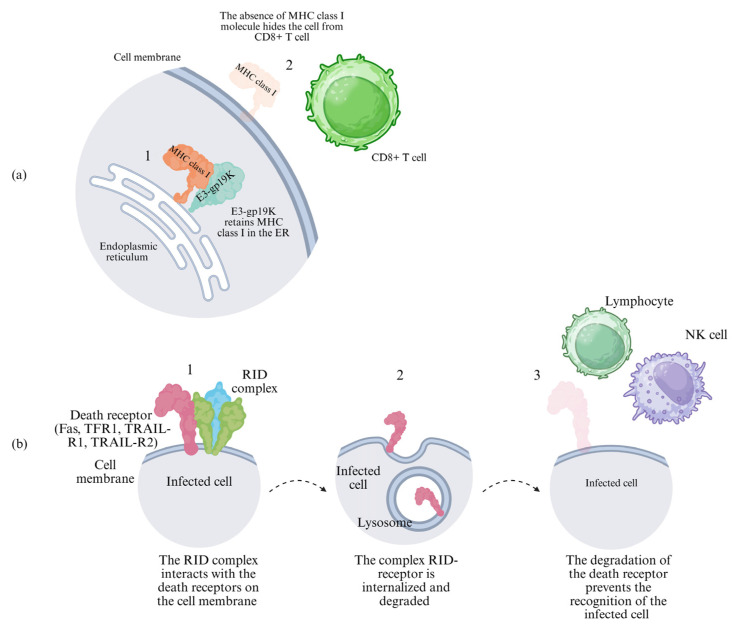
E3-gp19k and the RID complex promote immune evasion. (**a**) (1) E3-gp19k interacts with MHC class I molecules in the endoplasmic reticulum, preventing its exposure on the membrane. (2) Lack of MHC class I on the cell surface hides the infected cell from the CD8^+^ T cell. (**b**) (1) The adenoviral RID complex interacts with the host receptors on the cell surface, (2) causing them to be internalized and degraded. (3) The absence of receptors prevents the infected cell from being recognized by the immune cells.

E3-gp19K (also called E3-19K) binds β2-microglobulin, retaining MHC class I molecules in the endoplasmic reticulum. This prevents peptide loading and surface presentation to CD8^+^ T cells and NK cells [63,64] (Figure 5a), resulting in markedly reduced cytotoxic IFN-γ release. Notably, while E3-gp19K suppresses CD8^+^ T-cell recognition, its impact on T-cell infiltration in vivo remains unclear, as acute models have not shown significant differences following its deletion [46]; nevertheless, it could maintain an important role in the HAdV persistence in lymphoid cells [64,65]. Importantly, E3-gp19K’s affinity for HLA-A and HLA-B alleles differs from different HAdV and correlate MHC I cell -surface expression [66].

The E3-RID complex, a heterotrimeric transmembrane protein assembled from two RIDα (formerly called 10.4K) subunits and one RIDβ (formerly called 14.5K) subunit, is ubiquitous and highly conserved among HAdVs and serves as a central player in counteracting both apoptosis and inflammation, promoting the endocytosis and subsequent lysosomal degradation of key cell surface receptors [67]. Mechanistically, RID triggers clathrin-independent internalization of death receptors, including Fas (CD95), TRAIL-R1 (DR4), TRAIL-R2 (DR5), and TNFR1, followed by their sorting into lysosomes via Rab7-dependent maturation [68,69] (Figure 5b). This receptor degradation prevents ligand-induced signalling. For example, FasL or TRAIL binding is blocked, which inhibits caspase-8 activation and extrinsic apoptosis in infected cells [70,71]. Notably, the E3-6.7K protein cooperates with RID to ensure efficient degradation of TRAIL-R2, illustrating functional redundancy within the E3 cassette [68]. As observed with E3-gp19K, RID complex activity promotes persistent infection in lymphoid cells [72], interfering with the apoptotic signalling pathway. More recent insights from 2020 demonstrate that RID exerts broader anti-inflammatory effects by inhibiting TNF-α-triggered NF-κB and AP-1 signalling pathways [73]. A previous study showed that the inhibition of the NF-kB pathway is most likely caused by lack of surface receptors and does not involve direct activity of the RID complex on NF-kB [74].

In immunological contexts, these mechanisms enable robust HAdV immunoshadowing. Adenoviral RID complex inhibits TNF-α signalling, thereby avoiding the autocrine amplification loop that enhances the IL-6 and IL-1B production in macrophages and limits the inflammatory response during the infection. The TRAIL receptor degradation mediated by the RID complex reduces the sensitivity of infected cells to TRAIL exposed to the surface of NK cells, limiting the release of perforin/granzyme and slowing the viral clearance [69,75] (Figure 5b). For adaptive immunity, RID indirectly supports MHC class I evasion by prolonging the survival of infected cells, thereby allowing sustained suppression of IFN-γ secretion by CD8^+^ T cells [76]. The cited studies, despite strong validation through biochemical and functional assays, present the limit of the frequent use of transformed cell lines that offer microenvironments different from primary cells, where cytokines can modulate RID activity [73]; another layer of complexity arises from the transcriptional regulation of E3, since a 2025 study highlighted that host factors such as OCT4 can negatively regulate it by binding to the viral inverted terminal repeat (ITR). This repression may explain cell type- or subgroup-specific differences in RID efficacy and introduces a new controversy, because OCT4’s influence could limit RID activity in stem-like or cancerous cells [77].

Controversies persist regarding subgroup variability: RID from HAdV-C appears more potent against TRAIL receptors during persistent infections, whereas HAdV-B variants exhibit weaker NF-κB inhibition, potentially accounting for differences in disease severity [67]. Knowledge gaps include the impact of RID on emerging cytokines such as IL-27 that influence Th1 polarization [78], as well as its possible role in extracellular vesicle-mediated cytokine delivery during viral dissemination [79]. Future research should include the generation of CRISPR-edited RID mutant viruses for use in organoid models. These could be used to dissect cytokine dynamics (e.g., TNF-α/IL-6 ratios measured by ELISA) or combined with proteomics to identify novel host interactors such as ESCRT components involved in endosomal sorting [80]. Collectively, this expanded view positions the RID complex as a versatile “degradation hub” in HAdV virulence, offering clear translational potential for engineering RID-deficient vectors that enhance anti-tumour CD8^+^ T-cell responses in oncolytic therapies.

## 6. E4orf1 as a Pleiotropic Factor for Adenoviral Immune Evasion

The E4 genomic region produces up to seven different proteins from polycistronic transcripts. These include E4orf1 through E4orf6, as well as the spliced variant E4orf6/7 [81], which manipulate host signalling pathways and suppress antiviral responses [82]. Among these, E4orf1, E4orf3, and E4orf6 have been studied for their role in cell transformation and suppression of the antiviral response [83,84,85,86]. E4orf6 is best known for its tight functional partnership with E1B-55K, which together mediate p53 degradation via a Cullin5-based E3 ligase. However, E4orf6 also performs independent functions, such as CRM1-independent nuclear export of viral mRNAs, which helps sustain viral replication even when host protein synthesis is shut down [49].

**Figure 6:**
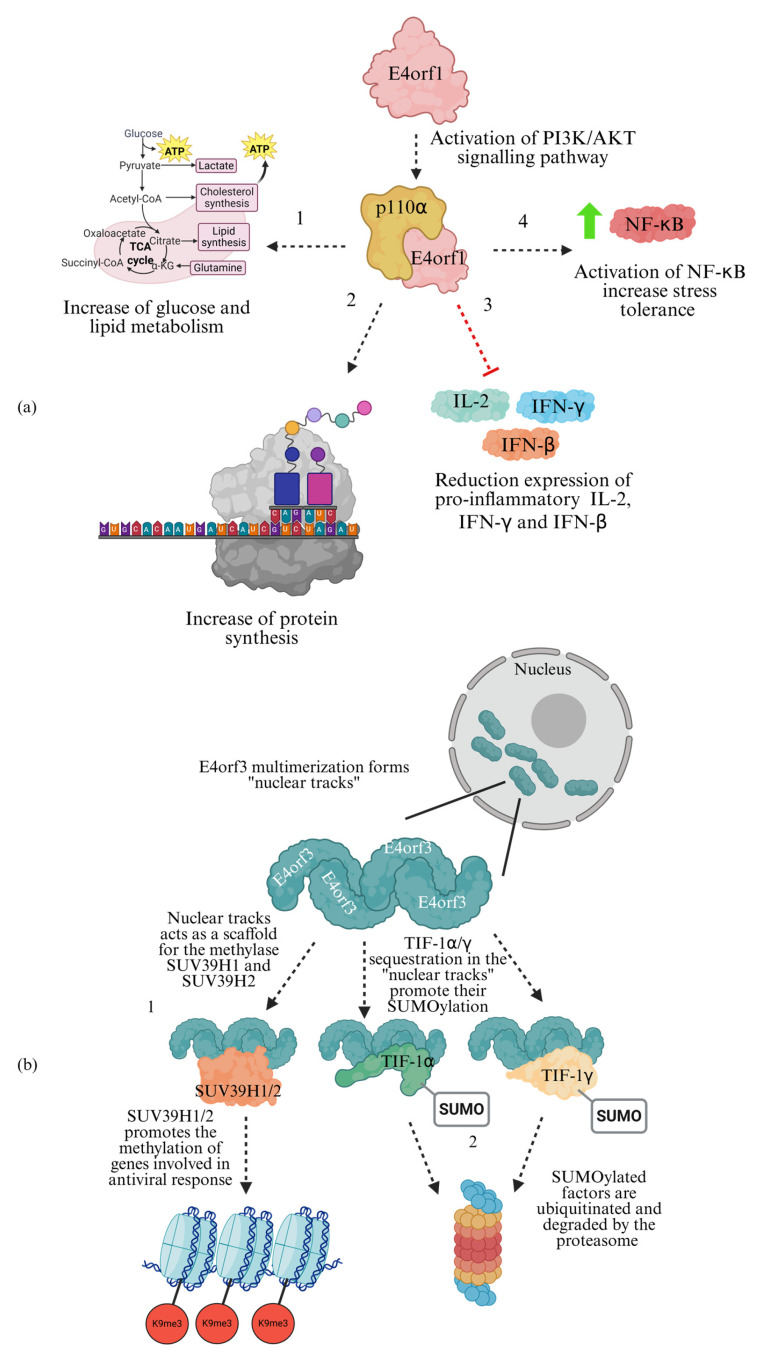
E4orf1 and E4orf3 also participate in adenoviral-mediated immune escape. (**a**) E4orf1 activation of PI3K/AKT signalling pathway has several effects: (1) increase in energy metabolism and (2) protein synthesis, (3) inhibition of immune response, and (4) activation of NF-κB to increase stress tolerance. (**b**) E4orf3 nuclear tracks act as a hub for multiple factors. (1) SUV39H1/2 interaction with nuclear tracks promotes the repression of antiviral genes. (2) Viral restriction factors are sequestered in the nuclear tracks, promoting their proteasome degradation.

E4orf1 activates the PI3K/AKT signalling pathway through interaction with the PI3K catalytic subunit p110α [83] (Figure 6a). Depending on experimental context, E4orf1-mediated AKT activation alters lipid and glucose metabolism and increases nuclear NF-κB levels via phosphorylation, supporting cell survival under polyploid conditions (DNA >4n) induced by infection [84,85]. More recently, E4orf1 was also shown to initiate a feedback loop in which NF-κB acts upstream of AKT, enhancing essential cellular activities such as protein synthesis through the phosphorylation of CDK2, to sustain cell cycle progression; it also acts upstream of NF-κB to survive in a stressed condition [86] (Figure 6a).

E4orf1 binds MYC, enhancing transcription of genes involved in metabolic pathways and nucleotide biosynthesis [87]. Despite activating NF-κB, E4orf1 represses innate immune responses through PI3K/AKT activation [88], abolishing induction of cytokines such as IL-2, IFN-γ, and IFN-β (Figure 6a). Although the interaction between E4orf1 and p110α (the 110 kDa catalytic subunit of the class IA PI3K enzyme) is conserved among HAdVs, interaction sites differ. E4orf1 from HAdV-C5 interacts with the domains RBD, L3H and K of p110α, leading to robust AKT activation [83], whereas E4orf1, from HAdV-4, interacts only via the RBD domain of p110α, resulting in weaker activation [83].

Upon activation, E4orf1 stimulates the PI3K pathway, reprogramming glucose metabolism in endothelial cells. These changes can indirectly modulate immune responses, potentially influencing cytokine production such as TNF-α in neighbouring immune cells.

## 7. E4orf3: Paving Nuclear Tracks for Immune Escape

E4orf3 is a suppressor of host immune response through the assembly of multimers that appear as filamentous structures called nuclear tracks [89] (Figure 6b), which trigger the reorganization of PML-NBs [90], promoting SUMOylation of viral restriction factors [91]. Unlike the E1B/E4orf6 complex, E4orf3 does not target MRN complex components for proteasomal degradation, but instead, it sequesters them into the nuclear tracks, thereby inactivating them [92,93] (Figure 6b). Formation of nuclear tracks via E4orf3 oligomerization promotes SUMOylation of the viral restriction factor TIF-1γ [94] (Figure 6b), leading to its degradation through an as-yet-unknown mechanism that appears independent of CRL2/CRL5 complexes [95]. Another antiviral factor, the ubiquitin ligase TIF-1α, is similarly sequestered within nuclear tracks and degraded [95,96] (Figure 6b), although it remains unclear whether this is mediated by E4orf3 enzymatic activity. Beyond destabilizing antiviral factors, E4orf3 nuclear tracks also serve as scaffolds for the H3-lysine 9 histone methyltransferases SUV39H1 and SUV39H2 [62,97] (Figure 6b), promoting trimethylation of H3K9 at specific loci, including MDM2, FAS, and TP53INP1 [62]. H3K9me3 deposition, in addition to prevent p53 recruitment, also represses the transcription of genes involved in the antiviral response, further dampening host immune response [62].

Key limitations of current research include the fact that most in vitro assays fail to reproduce the complex cytokine microenvironments found in vivo; for example, IL-6, secreted by macrophages, induces the deSUMOylation of PML-NBs, inactivating them [98] and could enhance the ability of E4orf3 to disrupt PML-NBs. In addition, controversies persist regarding the SUMO ligase activity of E4orf3 and its targeting of TIF-1γ; these effects appear to vary substantially by cell type. Overall, expanded analyses reveal extensive synergy between E4 proteins and the E3 region, enabling broad immunoshadowing.

## 8. Conclusions and Perspectives

HAdV immunoshadowing can be viewed as a highly coordinated “orchestra” of early viral proteins acting in concert. E1A initiates epigenetic reprogramming that suppresses innate pattern recognition receptors in macrophages, thereby reducing IL-1β and TNF-α production. E1B-55K reinforces this suppression through p53-dependent and SUMOylation-mediated repression of interferon-stimulated genes in plasmacytoid dendritic cells, blocking IFN-α/β release. E4orf1, E4orf3, and E4orf6 handle metabolic reprogramming and PML-body disruption to blunt TNF-α secretion from NK cells. Finally, E3 proteins complete the adaptive immune evasion program by retaining MHC class I molecules and degrading death receptors, collectively minimizing IFN-γ production by CD8^+^ T cells and IL-12 secretion by antigen-presenting cells [45,99]. While highlighting functional redundancies among adenoviral proteins, this synthesis also reveals critical gaps, such as subgroup-specific cytokine profiles (e.g., HAdV-E4 triggers stronger IL-6 responses in ocular infections [100] and potential sex differences, since females often mount heightened IFN-α responses [101], which may alter E3 protein efficacy). Another relevant point to consider is the wide use of transformed or cancerous cells, which frequently ignore the influence of the extracellular matrix on cytokine diffusion. Promising future directions include the integration of spatial transcriptomics to map cytokine gradients (such as IL-6 and CXCL10) directly within infected tissues, the development of small-molecule inhibitors of E3-mediated MHC class I down-regulation as adjunct therapies for disseminated disease, and longitudinal cytokine assays in transplant cohorts to resolve debates about the role of chronic immunoshadowing. Controversies also remain in vector applications; while E3-deleted constructs increase immunogenicity, they simultaneously raise the risk of excessive inflammation [102,103,104]. Taken together, this holistic perspective establishes human adenovirus as an outstanding model for studying viral–immune crosstalk and offers substantial translational potential for the design of next-generation precision virotherapies.

## Figures and Tables

**Table 1:** Selected viral proteins that act as molecular hubs to modulate host signalling pathways or interfere with epigenetic regulators, thereby repressing antiviral responses or promoting cell survival/proliferation. The table reports the direct interactions of viral proteins with host epigenetic regulators (e.g., DNMTs, HATs/HDACs, histone modifiers) that often feed into signalling pathways (e.g., IFN/JAK-STAT, NF-κB, TGFβ, innate immunity).

Virus	Viral Protein	Target	Pathway/Mechanisms	Reference
EBV	LMP1/2	JNK/AP-1; STAT3	Activation of the JNK/AP-1 and STAT3 signalling pathway	[9]
HBV	HBx	DNMT1; DNMT3A	Hypermethylation of IL-4R, USF1 promoters	[10]
HPV	E6; E7	DNMT1	Hypermethylation of IFNκ promoter	[11]
KSHV	LANA	STAT1	Inhibition of STAT1 phosphorylation	[12]
IAV	NS1	DNMT3B	Degradation of DNMT3B causes increase expression of JAK/STAT inhibitors	[13]
HSV-1	ICP27	STAT1	Inhibition of STAT1 phosphorylation	[14]
HIV	Tat	TAFII250	Inhibition of the TAFII250 HAT activity	[15]

## Data Availability

No new data were created or analyzed in this study.

## Abbreviations

The following abbreviations are used in this manuscript:

HAdV	Human adenovirus
EBV	Epstein–Barr virus
KSHV	Kaposi’s sarcoma-associated virus
HCMV	Human cytomegalovirus
HBV	Hepatitis B virus
HPV	Human papillomavirus
IAV	Influenza A virus
HIV	Human immunodeficiency virus
HAT	Histone acetyl-transferase
HDAC	Histone deacetylase
eRNA	Enhancer RNA
PML-NB	Promyelocytic leukaemia
SINE	Short interspersed nuclear elements
dsRNA	Double-stranded RNA
CRL	Cullin-RING ligase
IRG	Immune response gene
ISG	Interferon-stimulated genes

## References

[1] MacNeil K.M., Dodge M.J., Evans A.M., Tessier T.M., Weinberg J.B., Mymryk J.S. (2023). Adenoviruses in medicine: Innocuous pathogen, predator, or partner. Trends Mol. Med..

[2] Koski A., Kangasniemi L., Escutenaire S., Pesonen S., Cerullo V., Diaconu I., Nokisalmi P., Raki M., Rajecki M., Guse K. (2010). Treatment of cancer patients with a serotype 5/3 chimeric oncolytic adenovirus expressing GMCSF. Mol. Ther..

[3] Sampson A.T., Hlavac M., Gillman A.C.T., Douradinha B., Gilbert S.C. (2025). Developing the next-generation of adenoviral vector vaccines. Hum. Vaccin. Immunother..

[4] Gao J., Mese K., Bunz O., Ehrhardt A. (2019). State-of-the-art human adenovirus vectorology for therapeutic approaches. FEBS Lett..

[5] Russell S.J., Peng K.W., Bell J.C. (2012). Oncolytic virotherapy. Nat. Biotechnol..

[6] Goradel N.H., Mohajel N., Malekshahi Z.V., Jahangiri S., Najafi M., Farhood B., Mortezaee K., Negahdari B., Arashkia A. (2019). Oncolytic adenovirus: A tool for cancer therapy in combination with other therapeutic approaches. J. Cell. Physiol..

[7] Koski A., Karli E., Kipar A., Escutenaire S., Kanerva A., Hemminki A. (2013). Mutation of the fiber shaft heparan sulphate binding site of a 5/3 chimeric adenovirus reduces liver tropism. PLoS ONE.

[8] Ferrari R., Pellegrini M., Horwitz G.A., Xie W., Berk A.J., Kurdistani S.K. (2008). Epigenetic reprogramming by adenovirus e1a. Science.

[9] Wang Z., Luo F., Li L., Yang L., Hu D., Ma X., Lu Z., Sun L., Cao Y. (2010). STAT3 activation induced by Epstein-Barr virus latent membrane protein1 causes vascular endothelial growth factor expression and cellular invasiveness via JAK3 And ERK signaling. Eur. J. Cancer.

[10] Zheng D.L., Zhang L., Cheng N., Xu X., Deng Q., Teng X.M., Wang K.S., Zhang X., Huang J., Han Z.G. (2009). Epigenetic modification induced by hepatitis B virus X protein via interaction with de novo DNA methyltransferase DNMT3A. J. Hepatol..

[11] Yeo-Teh N.S.L., Ito Y., Jha S. (2018). High-Risk Human Papillomaviral Oncogenes E6 and E7 Target Key Cellular Pathways to Achieve Oncogenesis. Int. J. Mol. Sci..

[12] Lu F., Tsai K., Chen H.S., Wikramasinghe P., Davuluri R.V., Showe L., Domsic J., Marmorstein R., Lieberman P.M. (2012). Identification of host-chromosome binding sites and candidate gene targets for Kaposi’s sarcoma-associated herpesvirus LANA. J. Virol..

[13] Liu S., Liu L., Xu G., Cao Z., Wang Q., Li S., Peng N., Yin J., Yu H., Li M. (2019). Epigenetic Modification Is Regulated by the Interaction of Influenza A Virus Nonstructural Protein 1 with the De Novo DNA Methyltransferase DNMT3B and Subsequent Transport to the Cytoplasm for K48-Linked Polyubiquitination. J. Virol..

[14] Johnson K.E., Song B., Knipe D.M. (2008). Role for herpes simplex virus 1 ICP27 in the inhibition of type I interferon signaling. Virology.

[15] Weissman J.D., Brown J.A., Howcroft T.K., Hwang J., Chawla A., Roche P.A., Schiltz L., Nakatani Y., Singer D.S. (1998). HIV-1 tat binds TAF_II_250 and represses TAF_II_250-dependent transcription of major histocompatibility class I genes. Proc. Natl. Acad. Sci. USA.

[16] Pelka P., Ablack J.N., Fonseca G.J., Yousef A.F., Mymryk J.S. (2008). Intrinsic structural disorder in adenovirus E1A: A viral molecular hub linking multiple diverse processes. J. Virol..

[17] Komorek J., Kuppuswamy M., Subramanian T., Vijayalingam S., Lomonosova E., Zhao L.J., Mymryk J.S., Schmitt K., Chinnadurai G. (2010). Adenovirus type 5 E1A and E6 proteins of low-risk cutaneous beta-human papillomaviruses suppress cell transformation through interaction with FOXK1/K2 transcription factors. J. Virol..

[18] Glenewinkel F., Cohen M.J., King C.R., Kaspar S., Bamberg-Lemper S., Mymryk J.S., Becker W. (2016). The adaptor protein DCAF7 mediates the interaction of the adenovirus E1A oncoprotein with the protein kinases DYRK1A and HIPK2. Sci. Rep..

[19] Ferrari R., Berk A.J., Kurdistani S.K. (2009). Viral manipulation of the host epigenome for oncogenic transformation. Nat. Rev. Genet..

[20] Ferrari R., Gou D., Jawdekar G., Johnson S.A., Nava M., Su T., Yousef A.F., Zemke N.R., Pellegrini M., Kurdistani S.K. (2014). Adenovirus small E1A employs the lysine acetylases p300/CBP and tumor suppressor Rb to repress select host genes and promote productive virus infection. Cell Host Microbe.

[21] Ferrari R., Su T., Li B., Bonora G., Oberai A., Chan Y., Sasidharan R., Berk A.J., Pellegrini M., Kurdistani S.K. (2012). Reorganization of the host epigenome by a viral oncogene. Genome Res..

[22] Lutschg V., Boucke K., Hemmi S., Greber U.F. (2011). Chemotactic antiviral cytokines promote infectious apical entry of human adenovirus into polarized epithelial cells. Nat. Commun..

[23] Zemke N.R., Hsu E., Barshop W.D., Sha J., Wohlschlegel J.A., Berk A.J. (2023). Adenovirus E1A binding to DCAF10 targets proteasomal degradation of RUVBL1/2 AAA+ ATPases required for quaternary assembly of multiprotein machines, innate immunity, and responses to metabolic stress. J. Virol..

[24] Weinberg R.A. (1995). The retinoblastoma protein and cell cycle control. Cell.

[25] Liu X., Marmorstein R. (2007). Structure of the retinoblastoma protein bound to adenovirus E1A reveals the molecular basis for viral oncoprotein inactivation of a tumor suppressor. Genes Dev..

[26] Zemke N.R., Gou D., Berk A.J. (2019). Dedifferentiation by adenovirus E1A due to inactivation of Hippo pathway effectors YAP and TAZ. Genes Dev..

[27] Lohr K., Hartmann O., Schafer H., Dobbelstein M. (2003). Mutual interference of adenovirus infection and myc expression. J. Virol..

[28] Tworkowski K.A., Chakraborty A.A., Samuelson A.V., Seger Y.R., Narita M., Hannon G.J., Lowe S.W., Tansey W.P. (2008). Adenovirus E1A targets p400 to induce the cellular oncoprotein Myc. Proc. Natl. Acad. Sci. USA.

[29] Cantarella S., Vezzoli M., Carnevali D., Morselli M., Zemke N.R., Montanini B., Daussy C.F., Wodrich H., Teichmann M., Pellegrini M. (2024). Adenovirus small E1A directs activation of Alu transcription at YAP/TEAD- and AP-1-bound enhancers through interactions with the EP400 chromatin remodeler. Nucleic Acids Res..

[30] Ahmad S., Mu X., Yang F., Greenwald E., Park J.W., Jacob E., Zhang C.Z., Hur S. (2018). Breaching Self-Tolerance to Alu Duplex RNA Underlies MDA5-Mediated Inflammation. Cell.

[31] Cottrell K.A., Andrews R.J., Bass B.L. (2024). The competitive landscape of the dsRNA world. Mol. Cell.

[32] Tluckova K., Kaczmarek B., Salmazo A., Bernecky C. (2025). Mechanism of mammalian transcriptional repression by noncoding RNA. Nat. Struct. Mol. Biol..

[33] Yakovchuk P., Goodrich J.A., Kugel J.F. (2009). B2 RNA and Alu RNA repress transcription by disrupting contacts between RNA polymerase II and promoter DNA within assembled complexes. Proc. Natl. Acad. Sci. USA.

[34] Pelka P., Ablack J.N., Shuen M., Yousef A.F., Rasti M., Grand R.J., Turnell A.S., Mymryk J.S. (2009). Identification of a second independent binding site for the pCAF acetyltransferase in adenovirus E1A. Virology.

[35] Li X., Cho Y.S., Han Y., Zhou M., Liu Y., Yang Y., Zhuo S., Jiang J. (2025). The Hippo pathway effector YAP inhibits NF-κB signaling and ccRCC growth by opposing ZHX2. J. Biol. Chem..

[36] Berhane S., Areste C., Ablack J.N., Ryan G.B., Blackbourn D.J., Mymryk J.S., Turnell A.S., Steele J.C., Grand R.J. (2011). Adenovirus E1A interacts directly with, and regulates the level of expression of, the immunoproteasome component MECL1. Virology.

[37] Gregory S.M., Nazir S.A., Metcalf J.P. (2011). Implications of the innate immune response to adenovirus and adenoviral vectors. Future Virol..

[38] Carlin C.R. (2019). New Insights to Adenovirus-Directed Innate Immunity in Respiratory Epithelial Cells. Microorganisms.

[39] Federico S., Pozzetti L., Papa A., Carullo G., Gemma S., Butini S., Campiani G., Relitti N. (2020). Modulation of the Innate Immune Response by Targeting Toll-like Receptors: A Perspective on Their Agonists and Antagonists. J. Med. Chem..

[40] D’Anna S.E., Maniscalco M., Cappello F., Carone M., Motta A., Balbi B., Ricciardolo F.L.M., Caramori G., Stefano A.D. (2021). Bacterial and viral infections and related inflammatory responses in chronic obstructive pulmonary disease. Ann. Med..

[41] Ip W.H., Dobner T. (2020). Cell transformation by the adenovirus oncogenes E1 and E4. FEBS Lett..

[42] White E. (2006). Mechanisms of apoptosis regulation by viral oncogenes in infection and tumorigenesis. Cell Death Differ..

[43] Radke J.R., Grigera F., Ucker D.S., Cook J.L. (2014). Adenovirus E1B 19-kilodalton protein modulates innate immunity through apoptotic mimicry. J. Virol..

[44] Sato K., Ito K., Kohara H., Yamaguchi Y., Adachi K., Endo H. (1992). Negative regulation of catalase gene expression in hepatoma cells. Mol. Cell. Biol..

[45] Kato Y., Rice N., Pokrass M., Jeong J., Rodriguez R., Field J.J., Nowyhed H. (2024). Nonclinical characterization of ICVB-1042 as a selective oncolytic adenovirus for solid tumor treatment. Commun. Biol..

[46] Sparer T.E., Tripp R.A., Dillehay D.L., Hermiston T.W., Wold W.S., Gooding L.R. (1996). The role of human adenovirus early region 3 proteins (gp19K, 10.4K, 14.5K, and 14.7K) in a murine pneumonia model. J. Virol..

[47] Schreiner S., Wimmer P., Dobner T. (2012). Adenovirus degradation of cellular proteins. Future Microbiol..

[48] Pennella M.A., Liu Y., Woo J.L., Kim C.A., Berk A.J. (2010). Adenovirus E1B 55-kilodalton protein is a p53-SUMO1 E3 ligase that represses p53 and stimulates its nuclear export through interactions with promyelocytic leukemia nuclear bodies. J. Virol..

[49] Ip W.H., Tatham M.H., Krohne S., Gruhne J., Melling M., Meyer T., Gornott B., Bertzbach L.D., Hay R.T., Rodriguez E. (2023). Adenovirus E1B-55K controls SUMO-dependent degradation of antiviral cellular restriction factors. J. Virol..

[50] Baker A., Rohleder K.J., Hanakahi L.A., Ketner G. (2007). Adenovirus E4 34k and E1b 55k oncoproteins target host DNA ligase IV for proteasomal degradation. J. Virol..

[51] Dallaire F., Blanchette P., Groitl P., Dobner T., Branton P.E. (2009). Identification of integrin α3 as a new substrate of the adenovirus E4orf6/E1B 55-kilodalton E3 ubiquitin ligase complex. J. Virol..

[52] Gupta A., Jha S., Engel D.A., Ornelles D.A., Dutta A. (2013). Tip60 degradation by adenovirus relieves transcriptional repression of viral transcriptional activator EIA. Oncogene.

[53] Querido E., Blanchette P., Yan Q., Kamura T., Morrison M., Boivin D., Kaelin W.G., Conaway R.C., Conaway J.W., Branton P.E. (2001). Degradation of p53 by adenovirus E4orf6 and E1B55K proteins occurs via a novel mechanism involving a Cullin-containing complex. Genes Dev..

[54] Schreiner S., Burck C., Glass M., Groitl P., Wimmer P., Kinkley S., Mund A., Everett R.D., Dobner T. (2013). Control of human adenovirus type 5 gene expression by cellular Daxx/ATRX chromatin-associated complexes. Nucleic Acids Res..

[55] Schreiner S., Kinkley S., Burck C., Mund A., Wimmer P., Schubert T., Groitl P., Will H., Dobner T. (2013). SPOC1-mediated antiviral host cell response is antagonized early in human adenovirus type 5 infection. PLoS Pathog..

[56] Muncheberg S., Hay R.T., Ip W.H., Meyer T., Weiss C., Brenke J., Masser S., Hadian K., Dobner T., Schreiner S. (2018). E1B-55K-Mediated Regulation of RNF4 SUMO-Targeted Ubiquitin Ligase Promotes Human Adenovirus Gene Expression. J. Virol..

[57] Kolbe V., Ip W.H., Kieweg-Thompson L., Lang J., Gruhne J., Meyer T., Wilkens B., Schie M., Thunauer R., Schreiner S. (2022). Conserved E1B-55K SUMOylation in Different Human Adenovirus Species Is a Potent Regulator of Intracellular Localization. J. Virol..

[58] von Stromberg K., Seddar L., Ip W.H., Gunther T., Gornott B., Weinert S.C., Huppner M., Bertzbach L.D., Dobner T. (2023). The human adenovirus E1B-55K oncoprotein coordinates cell transformation through regulation of DNA-bound host transcription factors. Proc. Natl. Acad. Sci. USA.

[59] Seddar L., von Stromberg K., Bertzbach L.D., Grundhoff A., Dobner T., Ip W.H. (2025). Adenovirus E1B-55K regulates p53-dependent and -independent gene expression during infection. PLoS Pathog..

[60] Ip W.H., Bertzbach L.D., Schreiner S., Dobner T. (2025). Adenovirus E1B-55K interferes with cellular IκB kinase complex subunit proteins. Front. Immunol..

[61] Ozga A.J., Chow M.T., Lopes M.E., Servis R.L., Di Pilato M., Dehio P., Lian J., Mempel T.R., Luster A.D. (2022). CXCL10 chemokine regulates heterogeneity of the CD8^+^ T cell response and viral set point during chronic infection. Immunity.

[62] Soria C., Estermann F.E., Espantman K.C., O’Shea C.C. (2023). Author Correction: Heterochromatin silencing of p53 target genes by a small viral protein. Nature.

[63] McSharry B.P., Burgert H.G., Owen D.P., Stanton R.J., Prod’homme V., Sester M., Koebernick K., Groh V., Spies T., Cox S. (2008). Adenovirus E3/19K promotes evasion of NK cell recognition by intracellular sequestration of the NKG2D ligands major histocompatibility complex class I chain-related proteins A and B. J. Virol..

[64] Prasad V., Suomalainen M., Jasiqi Y., Hemmi S., Hearing P., Hosie L., Burgert H.G., Greber U.F. (2020). The UPR sensor IRE1α and the adenovirus E3-19K glycoprotein sustain persistent and lytic infections. Nat. Commun..

[65] Korner H., Burgert H.G. (1994). Down-regulation of HLA antigens by the adenovirus type 2 E3/19K protein in a T-lymphoma cell line. J. Virol..

[66] Fu J., Li L., Bouvier M. (2011). Adenovirus E3-19K proteins of different serotypes and subgroups have similar, yet distinct, immunomodulatory functions toward major histocompatibility class I molecules. J. Biol. Chem..

[67] Oliveira E.R.A., Bouvier M. (2019). Immune evasion by adenoviruses: A window into host-virus adaptation. FEBS Lett..

[68] Benedict C.A., Norris P.S., Prigozy T.I., Bodmer J.L., Mahr J.A., Garnett C.T., Martinon F., Tschopp J., Gooding L.R., Ware C.F. (2001). Three adenovirus E3 proteins cooperate to evade apoptosis by tumor necrosis factor-related apoptosis-inducing ligand receptor-1 and -2. J. Biol. Chem..

[69] Tollefson A.E., Toth K., Doronin K., Kuppuswamy M., Doronina O.A., Lichtenstein D.L., Hermiston T.W., Smith C.A., Wold W.S. (2001). Inhibition of TRAIL-induced apoptosis and forced internalization of TRAIL receptor 1 by adenovirus proteins. J. Virol..

[70] Elsing A., Burgert H.G. (1998). The adenovirus E3/10.4K-14.5K proteins down-modulate the apoptosis receptor Fas/Apo-1 by inducing its internalization. Proc. Natl. Acad. Sci. USA.

[71] Shisler J., Yang C., Walter B., Ware C.F., Gooding L.R. (1997). The adenovirus E3-10.4K/14.5K complex mediates loss of cell surface Fas (CD95) and resistance to Fas-induced apoptosis. J. Virol..

[72] McNees A.L., Garnett C.T., Gooding L.R. (2002). The adenovirus E3 RID complex protects some cultured human T and B lymphocytes from Fas-induced apoptosis. J. Virol..

[73] Monsalve F.A., Rojas A., Gonzalez I., Perez R., Anasco C., Romero J., Araya P., Santos L.S., Delgado-Lopez F. (2020). RID: Evaluation of the Possible Inhibiting Effect of the Proinflammatory Signaling Induced by TNF-*α* through NF-*κβ* and AP-1 in Two Cell Lines of Breast Cancer. Mediat. Inflamm..

[74] Delgado-Lopez F., Horwitz M.S. (2006). Adenovirus RIDαβ complex inhibits lipopolysaccharide signaling without altering TLR4 cell surface expression. J. Virol..

[75] Cardoso Alves L., Berger M.D., Koutsandreas T., Kirschke N., Lauer C., Sporri R., Chatziioannou A., Corazza N., Krebs P. (2020). Non-apoptotic TRAIL function modulates NK cell activity during viral infection. EMBO Rep..

[76] Gainey M.D., Rivenbark J.G., Cho H., Yang L., Yokoyama W.M. (2012). Viral MHC class I inhibition evades CD8^+^ T-cell effector responses in vivo but not CD8^+^ T-cell priming. Proc. Natl. Acad. Sci. USA.

[77] Ismail A.M., Saha A., Morrissey K.A., Lundquist D., Garcia E., Balne P., Cannon J.L., Chodosh J., Rajaiya J. (2025). OCT4 Negatively Regulates the Transcriptional Programming of the Early Region 3 Immune Evasion Genes of Human Adenovirus. bioRxiv.

[78] Owaki T., Asakawa M., Morishima N., Hata K., Fukai F., Matsui M., Mizuguchi J., Yoshimoto T. (2005). A role for IL-27 in early regulation of Th1 differentiation. J. Immunol..

[79] Saleem M., Chang C.W., Qadeer A., Asiri M., Alzahrani F.M., Alzahrani K.J., Alsharif K.F., Chen C.C., Hussain S. (2025). The emerging role of extracellular vesicles in viral transmission and immune evasion. Front. Immunol..

[80] Nachmias D., Frohn B.P., Sachse C., Mizrahi I., Elia N. (2025). ESCRTs—A multi-purpose membrane remodeling device encoded in all life forms. Trends Microbiol..

[81] Doorbar J. (2013). The E4 protein; structure, function and patterns of expression. Virology.

[82] Weitzman M.D. (2005). Functions of the adenovirus E4 proteins and their impact on viral vectors. Front. Biosci..

[83] Geng F., Kobayashi M., Lin Y., Gomez-Salinero J.M., Romano D., Kanyo J., Geng J., Liu Y., Ginsberg M., Shieh J.H. (2026). Adenovirus E4ORF1 activates isoform-specific phosphatidylinositol 3-kinase signaling in human endothelial cells. J. Biol. Chem..

[84] Almuzaini N., Moore M., Robert-Guroff M., Thomas M.A. (2022). Disruption of NBS1/MRN Complex Formation by E4orf3 Supports NF-κB That Licenses E1B55K-Deleted Adenovirus-Infected Cells to Accumulate DNA>4n. Microbiol. Spectr..

[85] Singhal G., Leo E., Setty S.K., Pommier Y., Thimmapaya B. (2013). Adenovirus E1A oncogene induces rereplication of cellular DNA and alters DNA replication dynamics. J. Virol..

[86] Moore M., Kong J., Akmel A., Thomas M.A. (2025). The adenovirus E4orf1 protein initiates a feedback loop involving insulin and growth factor receptors, AKT, and NF-κB, leading to abnormal DNA content in infected cells. PLoS Pathog..

[87] Thai M., Graham N.A., Braas D., Nehil M., Komisopoulou E., Kurdistani S.K., McCormick F., Graeber T.G., Christofk H.R. (2014). Adenovirus E4ORF1-induced MYC activation promotes host cell anabolic glucose metabolism and virus replication. Cell Metab..

[88] Sangare K., Helmold Hait S., Moore M., Hogge C., Hoang T., Rahman M.A., Venzon D.J., LaBranche C., Montefiori D., Robert-Guroff M. (2022). E4orf1 Suppresses E1B-Deleted Adenovirus Vaccine-Induced Immune Responses. Vaccines.

[89] Doucas V., Ishov A.M., Romo A., Juguilon H., Weitzman M.D., Evans R.M., Maul G.G. (1996). Adenovirus replication is coupled with the dynamic properties of the PML nuclear structure. Genes Dev..

[90] Patsalo V., Yondola M.A., Luan B., Shoshani I., Kisker C., Green D.F., Raleigh D.P., Hearing P. (2012). Biophysical and functional analyses suggest that adenovirus E4-ORF3 protein requires higher-order multimerization to function against promyelocytic leukemia protein nuclear bodies. J. Biol. Chem..

[91] Sohn S.Y., Hearing P. (2019). Mechanism of Adenovirus E4-ORF3-Mediated SUMO Modifications. mBio.

[92] Araujo F.D., Stracker T.H., Carson C.T., Lee D.V., Weitzman M.D. (2005). Adenovirus type 5 E4orf3 protein targets the Mre11 complex to cytoplasmic aggresomes. J. Virol..

[93] Stracker T.H., Carson C.T., Weitzman M.D. (2002). Adenovirus oncoproteins inactivate the Mre11-Rad50-NBS1 DNA repair complex. Nature.

[94] Sohn S.Y., Hearing P. (2016). The adenovirus E4-ORF3 protein functions as a SUMO E3 ligase for TIF-1γ sumoylation and poly-SUMO chain elongation. Proc. Natl. Acad. Sci. USA.

[95] Forrester N.A., Patel R.N., Speiseder T., Groitl P., Sedgwick G.G., Shimwell N.J., Seed R.I., Catnaigh P.O., McCabe C.J., Stewart G.S. (2012). Adenovirus E4orf3 targets transcriptional intermediary factor 1γ for proteasome-dependent degradation during infection. J. Virol..

[96] Bridges R.G., Sohn S.Y., Wright J., Leppard K.N., Hearing P. (2016). The Adenovirus E4-ORF3 Protein Stimulates SUMOylation of General Transcription Factor TFII-I to Direct Proteasomal Degradation. mBio.

[97] Vink E.I., Zheng Y., Yeasmin R., Stamminger T., Krug L.T., Hearing P. (2015). Impact of Adenovirus E4-ORF3 Oligomerization and Protein Localization on Cellular Gene Expression. Viruses.

[98] Ohbayashi N., Kawakami S., Muromoto R., Togi S., Ikeda O., Kamitani S., Sekine Y., Honjoh T., Matsuda T. (2008). The IL-6 family of cytokines modulates STAT3 activation by desumoylation of PML through SENP1 induction. Biochem. Biophys. Res. Commun..

[99] Locatelli M., Faure-Dupuy S. (2023). Virus hijacking of host epigenetic machinery to impair immune response. J. Virol..

[100] Van Gelder R.N., Akileswaran L., Nakamichi K., Stroman D. (2022). Molecular and Clinical Characterization of Human Adenovirus E4-Associated Conjunctivitis. Am. J. Ophthalmol..

[101] Pujantell M., Altfeld M. (2022). Consequences of sex differences in Type I IFN responses for the regulation of antiviral immunity. Front. Immunol..

[102] Ginsberg H.S., Lundholm-Beauchamp U., Horswood R.L., Pernis B., Wold W.S., Chanock R.M., Prince G.A. (1989). Role of early region 3 (E3) in pathogenesis of adenovirus disease. Proc. Natl. Acad. Sci. USA.

[103] Ilan Y., Droguett G., Chowdhury N.R., Li Y., Sengupta K., Thummala N.R., Davidson A., Chowdhury J.R., Horwitz M.S. (1997). Insertion of the adenoviral E3 region into a recombinant viral vector prevents antiviral humoral and cellular immune responses and permits long-term gene expression. Proc. Natl. Acad. Sci. USA.

[104] Lesokhin A.M., Delgado-Lopez F., Horwitz M.S. (2002). Inhibition of chemokine expression by adenovirus early region three (E3) genes. J. Virol..

